# Complete mitochondrial genome of the planthopper *Orthopagus splendens* (Germar, 1830) (Hemiptera: Fulgoromorpha: Dictyopharidae)

**DOI:** 10.1080/23802359.2021.1964400

**Published:** 2021-08-16

**Authors:** Yan-li Zheng, Thierry Bourgoin, Lin Yang, Xiang-Sheng Chen, Xu-Qiang Luo, Guang-Jie Luo

**Affiliations:** a Institute of Entomology, Guizhou University, Guiyang, PR China; b The Provincial Key Laboratory for Agricultural Pest Management of Mountainous Region, Guiyang, PR China; c School of Geography and Resources, Guizhou Education University, Guiyang, PR China; d Guizhou Provincial Key Laboratory of Geographic State Monitoring of Watershed, PR China; e Institut Systématique, Evolution, Biodiversité (ISYEB), MNHN-CNRS-Sorbonne Université-EPHE-Univ. Antilles, Museum National d'Histoire Naturelle, Paris, France

**Keywords:** *Orthopagus splendens*, Dictyopharidae, mitogenome, phylogeny

## Abstract

The first complete mitochondrial genome of a dictyopharid planthopper, *Orthopagus splendens* (Germar, 1830) (Hemiptera: Fulgoroidea: Dictyopharidae) is sequenced. The 15,349 bp long complete mitogenome contains 13 protein-coding genes (PCGs), 22 *tRNA* genes, two rRNA genes, and 1 A + T-rich region with an arrangement identical to that observed in most insect mitogenomes (GenBankNo. MW441850). All PCGs start with ATN, and end with TAN or single T (*nad1*, *nad5*, and *atp6*). A phylogenetic analysis places *O. splendens* as sister to Fulgoridae confirming a sister relationship between Dictyopharidae and Fulgoridae.

The planthoppers of the family Dictyopharidae Spinola, 1839 (Hemiptera: Fulgoromorpha), currently groups 740 species in 160 genera (Bourgoin 2020). Divided into two subfamilies and 19 tribes, the species *Orthopagus splendens* (Germar, 1830) belongs to the Dictypharinae Spinola, 1839, Orthopagini Emeljanov 1983 (Muir 1923; Metcalf 1946; Song et al. 2018; Bourgoin 2020). We report here the first complete mitochondrial genome (mitogenome) for this planthopper family.

Mitogenome DNA was extracted from a single female specimen collected from Xinliangtian Village, Guiding County, Guizhou province, China (107°23′E, 26°57′N) on the 11 August 2020. after DNA was extracted from the hind legs, a specimen was deposited at the Institute of Entomology, Guizhou University (Xiangsheng Chen, chenxs3218@163.com) under the voucher number GUGC-20200811-Y1. The mitogenome sequences were obtained by next-generation sequencing method (Illumina HiSeq X 10), assembled using Geneious version 10.2.2 (Kearse et al. 2012) , annotated, and conducted using MITOZ (Meng et al. 2019) and deposited in GenBank, accession number MW441850. IQtree (Kumar et al. 2016) was used for phylogenetic analyses and to construct the maximum likelihood (ML) tree based on 13 PCGs, 22 *tRNA*, and two rRNA of 23 species (including four outgroups from Cicadelloidea and Cicadoidea), and 18 related planthopper species from different Fulgoroidea families.

The complete mitogenome of *O. splendens* is 15,349 bp in length and consists of 13 protein-coding genes (PCGs), two ribosomal *RNA* genes (*rRNAs*), 22 transfer *RNA* genes (*tRNAs*), and one large non-coding region (D-loop: [A + T]-rich region). The D-loop part is 1181 bp, taking place between *12S* rRNA and *trnI*. The overall bases composition is A: 30.2%, T: 47.2%, C: 14.6%, and G: 8%. AT skew ((A − T)/(A + T)) and GC skew ((G − C)/(G + C)) are 0.22, −0.294, respectively. All the 13 PCGs started with ATN, ended with TAN or a single T (*nad1*, *nad5*, and *atp6*) residue. The length of 22 tRNA ranged from 59 bp(*trnS*) to 70 bp (*trnK*). Genes of *16S rRNA* and *12S rRNA* are 1177 and 729 bp, respectively.

The phylogenetic tree obtained confirms the monophyly of Fulgoroidea and most nodes are recovered with the higher ultrafast bootstrap (UFB = 100). As expected from both morphological (Emeljanov 1979; Song et al. 2018) and molecular analyses (Urban and Cryan 2009; Song and Liang 2013), the dictyopharid species appears in a sister relationship with Fulgoridae, both in an unexpected clade sister to Achilidae: (Achilidae+(Dictyopharidae + Fulgoridae)). While already previously observed by Song and Liang (2013) and Wang et al. (2019), it is probably due to a limited sampling effect.

## Data Availability

The data supporting the findings of this study are openly available in the National Center for Biotechnology Information (NCBI) at https://www.ncbi.nlm.nih.gov, accession number MW441850. The associated BioProject, SRA, and Bio-Sample numbers are PRJNA726041, SRS8798664, and SAMN18920653, respectively. Maximum likelihood tree based on 23 complete mitochondrial genome sequences. Fulgoromorpha: 19 in-group species and four outgroup species: two in Cicadellidae and two in Cicadidae.
